# Delayed Diagnosis of Vertebrobasilar Artery Stenosis Developed During Follow-Up of Two Patients With Pediatric-Onset Moyamoya Disease

**DOI:** 10.7759/cureus.87805

**Published:** 2025-07-13

**Authors:** Bongguk Kim, Shoko Hara, Motoki Inaji, Hiroyuki Akagawa, Taketoshi Maehara

**Affiliations:** 1 Department of Neurosurgery, Institute of Science Tokyo, Tokyo, JPN; 2 Institute for Comprehensive Medical Sciences, Tokyo Women’s Medical University, Tokyo, JPN

**Keywords:** cardiovascular genetics, disease progression, moyamoya disease (mmd), pediatric disease, rnf213, vertebral basilar artery

## Abstract

Moyamoya disease is characterized by bilateral stenosis of the terminal portions of the internal carotid arteries, whereas stenotic lesions in vertebrobasilar artery stenosis are reported less frequently. Moreover, de novo stenosis in these vessels during follow-up is exceptionally uncommon and rarely reported in the literature. Herein, we report two patients with pediatric-onset moyamoya disease who developed vertebrobasilar artery stenosis during follow-up after initial surgery. The first patient received initial surgery at the age of four years and demonstrated progression of bilateral vertebral artery stenosis at age 10 that eventually led to postoperative cerebellar ischemic stroke. The second case was diagnosed as moyamoya disease at the age of 15 years and developed basilar artery stenosis with shrinkage of the outer diameter at age 28. Vertebrobasilar stenosis of both patients was undiagnosed when it first appeared, and their diagnosis was delayed for years. The first patient carried the *RNF213* p.R4810K variant, the susceptibility variant of moyamoya disease in the Asian population, but the second patient did not have any variant in this gene. These cases suggested that, although rare, vertebrobasilar artery stenosis can develop or progress during follow-up in patients with moyamoya disease, so careful assessment of the vertebrobasilar arteries, as well as the arteries of the circle of Willis, is recommended during follow-up. While variants in* RNF213* are known to affect vascular structure and clinical prognosis, the inconsistent results of the *RNF213* gene variant in our patient suggested that this gene variant is not sufficient to explain vertebrobasilar artery stenosis in this disease. Further accumulation of cases is required to unravel the pathophysiology of this rare condition.

## Introduction

Moyamoya disease is a rare cerebrovascular disorder characterized by progressive stenosis of the terminal portions of the bilateral internal carotid arteries (ICAs), leading to the development of compensatory collateral vessels known as moyamoya vessels [1]. The disease predominantly affects children and young adults in East Asian countries, including Japan, and the incidence in these countries was 10 times higher than in Western countries for adults (0.54 vs. 0.047-0.086 per 100,000, respectively) and 20 times higher for children (5.3 vs. 0.39 per 100,000, respectively) [2].* *The *RNF213* p.R4810K variant, the so-called founder variant of this gene, has been identified as a major susceptibility gene in East Asian countries. This gene is now known to be associated with systemic arterial stenoses beyond the circle of Willis, such as the coronary artery, iliac artery [3,4], renal artery [5], pulmonary artery [6], as well as non-moyamoya intracranial arterial stenosis. A Korean study has shown that when evaluating non-moyamoya disease patients with ischemic stroke in middle cerebral artery (MCA) regions, all intracranial arteries (ICA, MCA, and basilar artery (BA)) of patients with the *RNF213* variant were smaller than patients without this variant [7]. Recent MRI studies have demonstrated that stenotic lesions in moyamoya disease are associated with "negative remodeling," characterized by shrinkage of the outer diameter of the affected arteries on heavy T2-weighted imaging [8]. In advanced stages of this disease, posterior cerebral arteries (PCAs) are also involved and stenosed, as well as ICAs, MCAs, and anterior cerebral arteries (ACAs). While no preventive strategy for progressive stenosis currently exists, revascularization surgery improves hemodynamic compromise induced by intracranial arterial stenosis.

Although disease progression can sometimes involve PCAs, stenosis extending to the posterior portion of the intracranial arteries, such as vertebral arteries (VAs) or BAs, is rare (1.5-3.0%) [9]. Moreover, de novo stenosis in these vessels during follow-up is exceptionally uncommon and rarely reported in the literature. Some researchers hypothesized that the difference of embryological origin may explain why stenosis of posterior circulation is rare in moyamoya disease (i.e., anterior circulation arteries (ACA, MCA, and P2-P4 portions of PCA) derived from the primitive ICA while VAs, BA, and P1 portion of PCA derived from the longitudinal neural dorsal plexus and intersegmental arteries) [10]. However, the pathophysiology of VA/BA stenosis in moyamoya disease, or its relationship to *RNF213*, is not well established.

Herein, we report two cases of pediatric-onset moyamoya disease with delayed diagnosis of VA/BA stenosis that developed during follow-up after initial revascularization surgery. We also performed genetic analysis focusing on the *RNF213* in these patients to see the possible linkage between the *RNF213* variant and VA/BA stenosis.

## Case presentation

Case 1

A four-year-old girl with a family history of her grandmother’s moyamoya disease, but no significant past medical history, developed headaches and transient left hemiparesis. She was diagnosed with moyamoya disease and underwent bilateral combined revascularization surgery at another hospital. Afterwards, she remained asymptomatic until the age of 10, when she began experiencing repetitive transient visual disturbances, dysarthria, and right lower limb weakness. She was referred to our department at age 11 for further treatment.

When she was referred to our institution, she was free of neurological deficit and visible deformity, suggesting an underlying disease of moyamoya syndrome (Down syndrome, neurofibromatosis type 1, or other genetic diseases). Her laboratory results were within normal limits, including thyroid hormones, and her antithyroid autoantibodies were negative.

**Figure 1:**
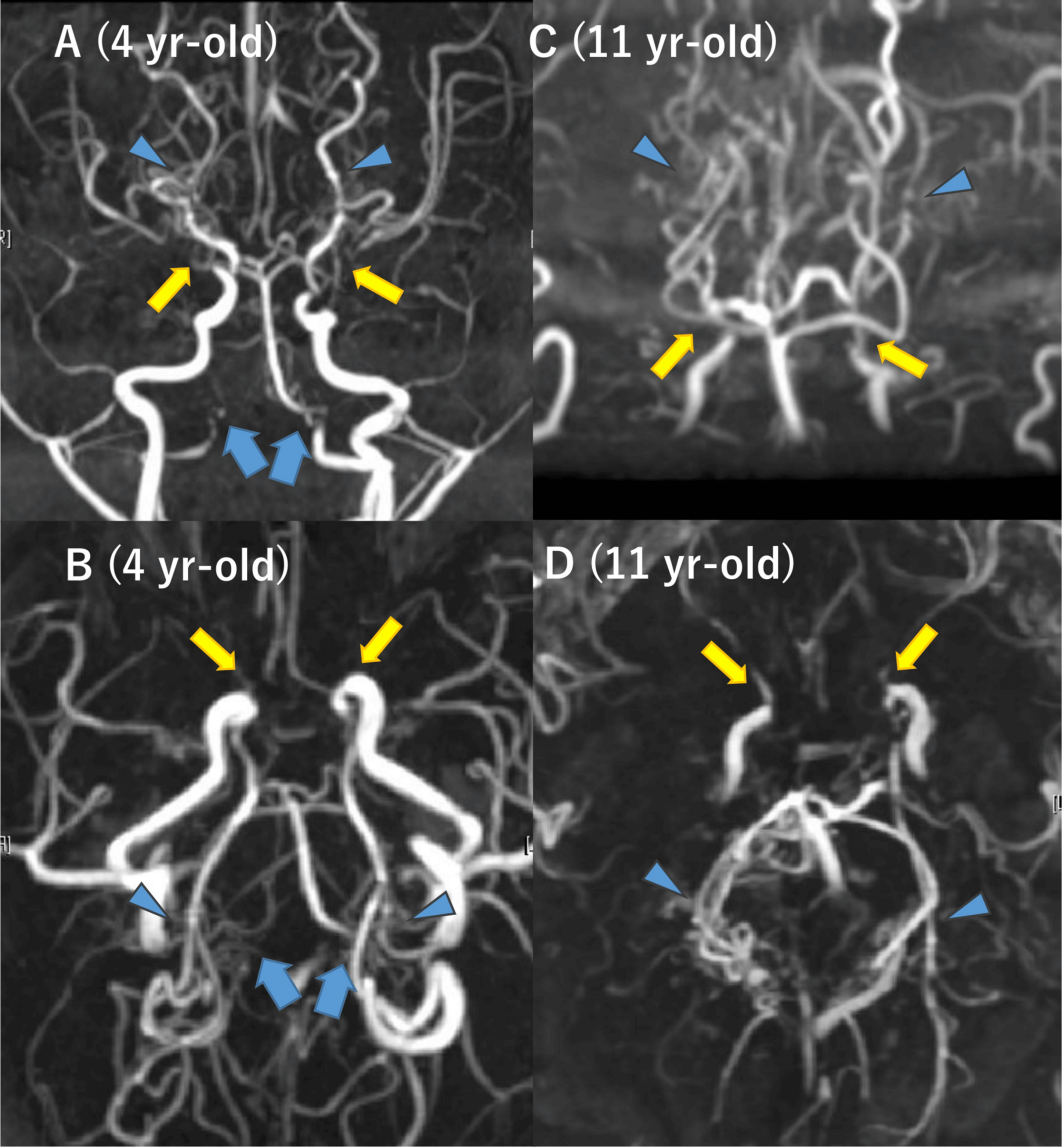
Initial and current magnetic resonance angiography of case 1. At age four (A and B), bilateral internal carotid artery (ICA) stenosis was observed (yellow arrows), and bilateral vertebral artery (VA) stenosis was suspected (blue arrows). Posterior cerebral arteries (PCAs) were clearly visualized without significant stenosis (blue arrowheads). At age 11 (C and D), in addition to bilateral ICA stenosis, bilateral PCA stenosis that did not exist at age four was also noted (blue arrowheads). The bilateral VAs were outside the imaging range and could not be evaluated (blue arrows).

**Figure 2:**
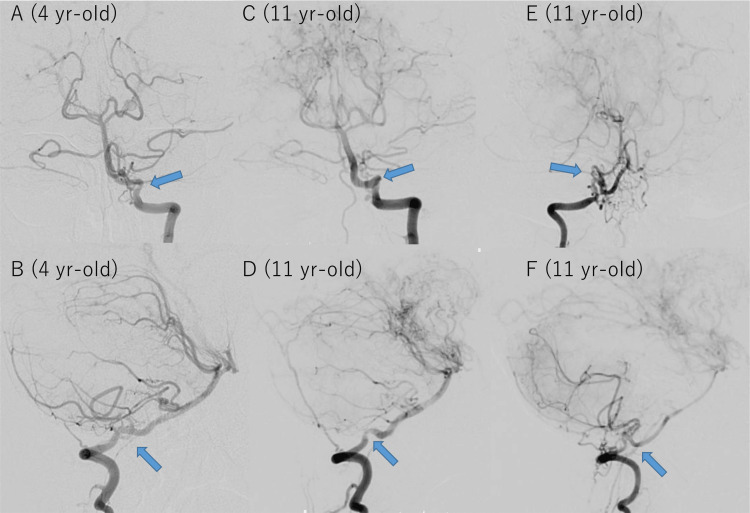
Digital subtraction angiography of the initial and current presentation of case 1. At age four, left vertebral artery (VA) angiography revealed tortuosity in the V4 segment, but no definite stenosis (A and B; indicated by blue arrows). The right VA was not imaged. At age 11, mild stenosis was observed in the left V4 segment, which had progressed from the images at age four. The right V4 segment showed stenosis at the tortuous portion along with proliferation of moyamoya vessels (C-F; stenosis indicated by blue arrows).

At age four, magnetic resonance angiography (MRA) revealed severe stenosis of the bilateral terminal ICAs and proliferation of moyamoya vessels (Figures 1A, 1B). Bilateral stenosis of the V4 segments of VAs was suspected, but the treating neurosurgeons did not recognize it at the time. Subsequently, V4 segments were not included in the acquisition range of MRA, leaving the clinical course of V4 segments unknown (Figures 2C, 2D). No apparent stenotic lesions were observed in the PCAs at age 4, but progressive stenosis of the ICAs and PCAs was noted during follow-up (Figures 1C, 1D).

**Figure 3:**
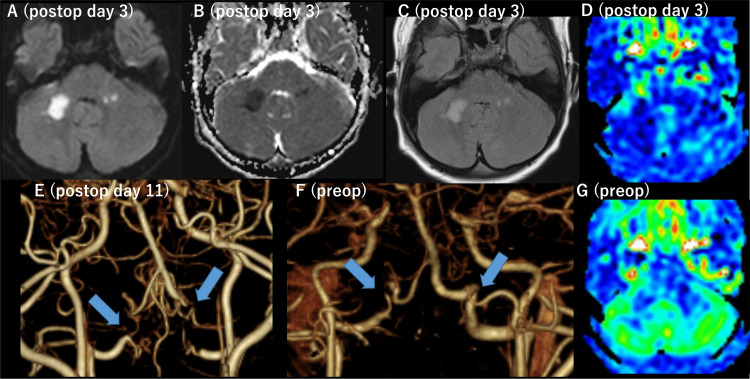
Preoperative and postoperative magnetic resonance imaging and computed tomography angiography of case 1. On postoperative day three, acute ischemic stroke was observed in bilateral middle cerebellar peduncles, appearing as high-intensity lesions on diffusion-weighted imaging (A), low-intensity lesions on apparent diffusion coefficient (B), and high-intensity lesions on fluid attenuated inversion recovery (C). Cerebral blood flow map of the arterial spin labeling method revealed diffuse hypoperfusion of the bilateral cerebellar hemispheres (D) that was not observed before the surgery (G). Postoperative CTA conducted after the onset of cerebellar ischemic stroke revealed occlusion of bilateral vertebral arteries (VAs) at the V4 segment (E, arrows) that clearly progressed from the preoperative CT angiography (F).

On digital subtraction angiography (DSA) at age 4, stenosis of the left VA was not evident, but the right VA was not evaluated; thus, whether stenosis suspected by MRA truly existed at this time remains unknown (Figures 2A, 2B). At age 11, mild stenosis of the bilateral V4 segment was noted, and progression of the left VA stenosis was suggested. The stenosed portion of the right V4 segment was surrounded by fine collateral vessels resembling basal moyamoya vessels (Figures 2C-1F). Main trunks of bilateral PCAs were not visualized, and only cortical arteries of the right PCA territory were slightly opacified via leptomeningeal anastomosis from the right ICA. Cerebral blood flow studies using arterial spin labeling MRI [11] demonstrated hypoperfusion and delayed perfusion in the bilateral parieto-occipital lobes but no hypoperfusion in the cerebellum (Figure 3D). Ivy signs were observed in the bilateral parieto-occipital lobes, but not in the cerebellum. Surgical revascularization for parieto-occipital regions was deemed necessary, and encephalo-duro-pericranial-synangiosis [12] was scheduled. While bilateral V4 segment stenosis was observed at the second DSA in our institution, we did not plan any intervention for these lesions because she lacked symptoms originating from these stenosis, and cerebellar perfusion seems intact.

One month before the surgery, aspirin (80 mg/day, 1.97 mg/kg/day) and cilostazol (50 mg/day, 1.23 mg/kg/day) were initiated. Aspirin was discontinued one week before the surgery, while cilostazol was continued. Intravenous fluids of 1000 mL/day (1.03 mL/kg/h) were administered the day before surgery and continued at 1500 mL/day (1.54 mL/kg/h) postoperatively. The surgery and immediate perioperative courses were uneventful; she interacted well with her mother and showed a good appetite until postoperative day two. However, on postoperative day three, she suddenly developed decreased speech and appetite. MRI revealed acute bilateral cerebellar ischemic stroke and diffuse hypoperfusion of the cerebellum (Figures 3A-3D), and her decreased speech was regarded as cerebellar mutism. Postoperative MRA showed poor visualization of the bilateral VAs at the V4 segments, and computed tomography angiography (CTA) confirmed occlusion at the same locations (Figures 3F, 3G); these sudden occlusions of the bilateral V4 segments confirmed bilateral VA stenosis induced by moyamoya disease. Intravenous fluids, cilostazol, and intensive rehabilitation led to the gradual recovery of her symptoms. The patient was discharged home on postoperative day 32, and subsequent follow-up at the outpatient clinic revealed near-complete resolution of her symptoms. Her follow-up MRA, including the lower portion of VA, showed stable BA stenosis.

Genetic analysis of* RNF213 *(details described in a previous study [13]) revealed a heterozygous p.R4810K variant, but no other rare variants were found.

Case 2

A 15-year-old female, whose father and paternal grandmother had moyamoya disease and who had no significant past medical history, developed a small ischemic stroke in the left corona radiata and was diagnosed with moyamoya disease at another hospital. She was prescribed aspirin and followed up without surgery at that facility. At age 20, she developed involuntary movements of the left upper extremity and was referred to our hospital. She received bilateral indirect revascularization targeting frontoparietal areas (encephalo-duro-arterio-synangiosis). After surgery and discontinuation of aspirin, her involuntary movements disappeared, and she remained well for years, experiencing only infrequent transient paresthesia in her hands when she laughed a lot. At age 29, because of the retirement of her treating neurosurgeon, she was referred to a local institute. However, at age 30, she experienced transient dizziness, and MRA obtained at the local institute revealed marked stenosis in the BA. She was then referred to our hospital.

When she revisited our hospital, she was free of neurological deficit and visible deformity, suggesting an underlying disease of moyamoya syndrome (Down syndrome, neurofibromatosis type 1, or other genetic diseases). Her laboratory results were within normal limits, including thyroid hormones, and her antithyroid autoantibodies were negative. Systemic vascular screening using coronary and aorta computed tomography arteriography revealed no stenosis in cardiac, renal, and other systemic arteries. Cardiac ultrasound revealed no cardiac abnormalities and normal cardiac function.

**Figure 4:**
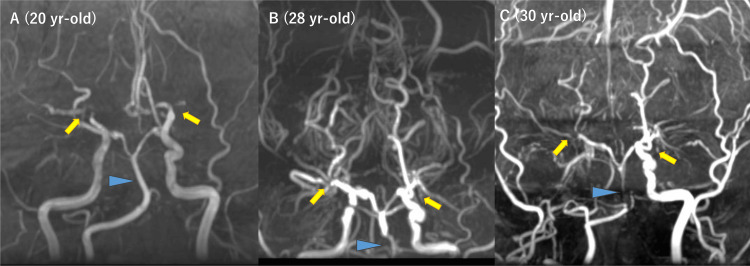
Initial and follow-up magnetic resonance angiography of case 2. At age 20 (A), bilateral internal carotid arteries (ICAs) were stenosed (yellow arrows), while the basilar artery (BA) was normal (blue arrowhead). At age 28 (B), though it was not suspected at that time, a retrospective review revealed BA stenosis that did not exist at age 20. By age 30 (C), the progression of BA stenosis was evident and had progressed compared to previous studies.

**Figure 5:**
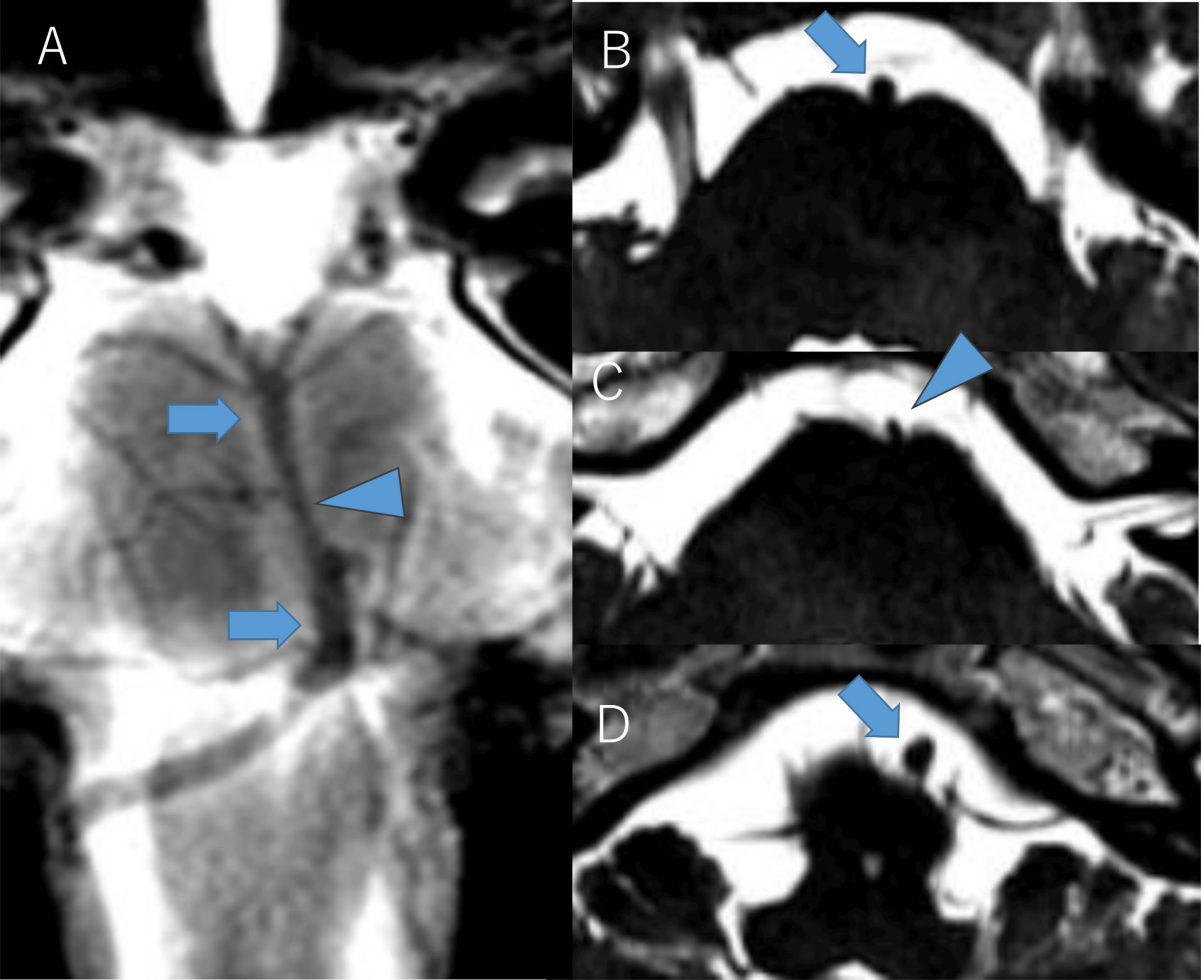
MRI showing shrinkage of the outer diameter in basilar artery (BA) of case 2. Basi-parallel anatomical scanning (A) and heavy T2-weighted images (B-D) acquired to evaluate the outer diameter of BA revealed that the outer diameter of the stenosed portion of BA was smaller than the non-stenosed portion. The outer diameters of the proximal part, stenosed part, and the distal part of BA were measured as 3.0 mm (B, blue arrow), 1.9 mm (C, blue arrowhead), and 3.8 mm (D, blue arrow), respectively, which highlighted the shrinkage of the outer diameter of the stenosed portion.

**Figure 6:**
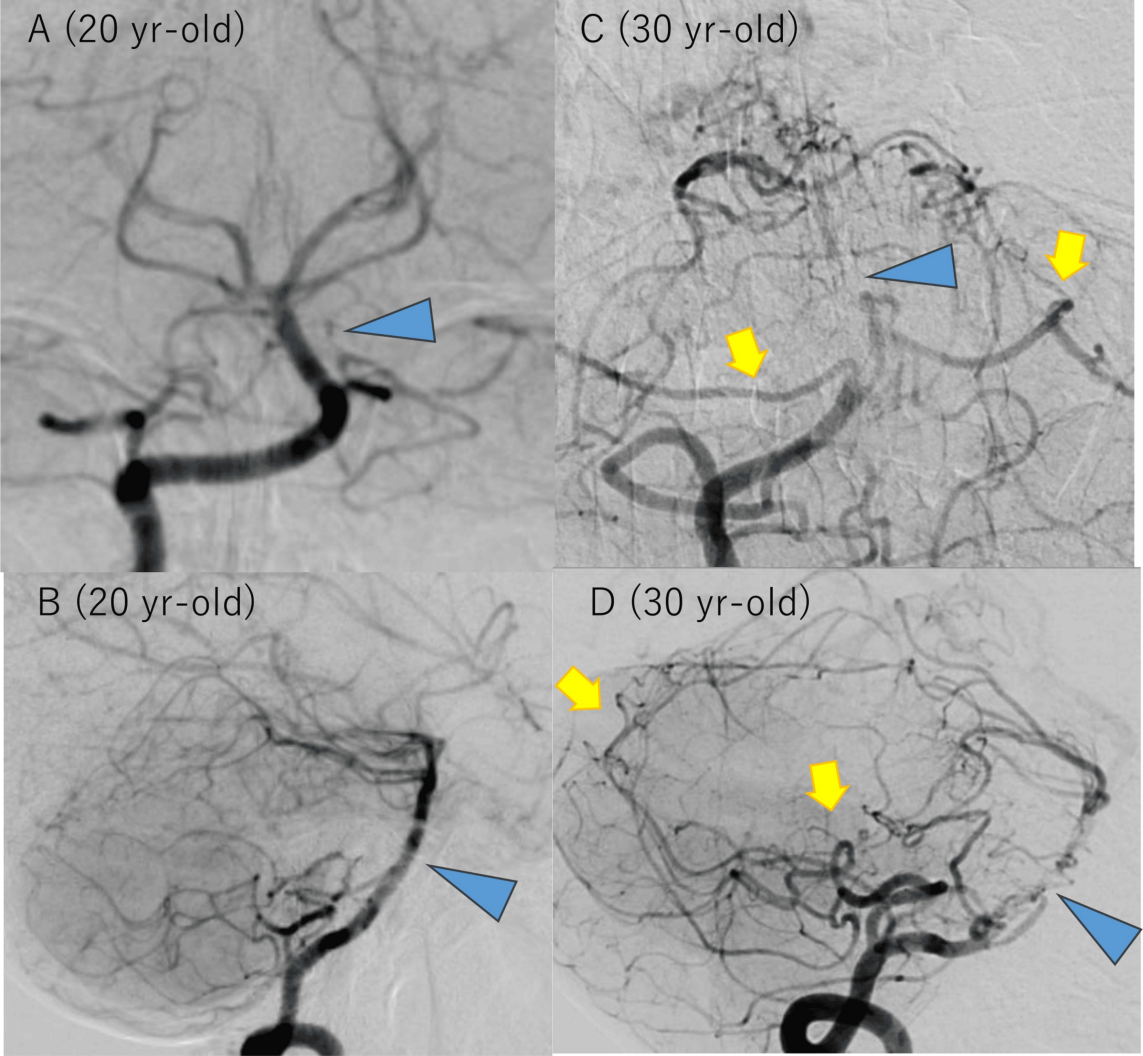
Initial and follow-up digital subtraction angiography of case 2. At age 20 (A and B), no stenosis was observed in the basilar artery (BA, blue arrowheads). At age 30 (C and D), in addition to severe BA stenosis that did not exist at age 20 (blue arrowheads), and posterior inferior cerebellar arteries (yellow arrows) were dilated and visualized well compared to the images at age 20. The distal portion of BA was visualized via leptomeningeal collateral flow from the developed posterior inferior cerebellar arteries to superior cerebellar arteries (yellow arrows).

On initial MRA at age 20, severe stenosis of the bilateral terminal ICAs was noted, but BA appeared normal (Figure 4A). At age 28, retrospective observation identified that BA stenosis had already presented, but the treating neurosurgeon and radiologists did not notice it at that time, probably because the lower end of the acquisition range started from the stenosed portion of BA and the neurosurgeon and radiologists only focused on anterior circulation and did not pay much attention to BA (Figure 4B). At age 30, when she was referred to our institution again, BA stenosis was apparent, with decreased signal intensity in the distal part of the stenosis (Figure 4C). Basi-parallel anatomical scanning (Figure 5A) and heavy T2-weighted imaging (Figures 5B-5D) demonstrated that the stenosed portion of BA had a reduced outer diameter. Conventional imaging revealed no ischemic stroke in the cerebellum or brainstem. DSA confirmed severe BA stenosis, with perfusion to the distal part maintained via leptomeningeal anastomosis from the posterior inferior cerebellar arteries to the anterior inferior cerebellar arteries and superior cerebellar arteries (Figure 6). ^15^O-gas PET [14] showed misery perfusion in the right parieto-occipital lobe, but cerebellar hemodynamics (cerebral blood flow, cerebral blood volume, oxygen extraction fraction, and cerebral metabolic ratio of oxygen) were within the normal range. Ivy signs were observed in the bilateral frontal, parietal, and right occipital lobes, but not in the cerebellum.

After discussing the condition with the patient, we decided to avoid surgical intervention for the BA stenosis due to the high associated risks and to manage her with antiplatelet therapy using cilostazol (200 mg/day). Misery perfusion of the right parieto-occipital lobe was treated with indirect revascularization (encephalo-duro-pericranial-synangiosis), which resolved after the surgery. Her follow-up MRA, including the lower portion of BA/VA, showed stable BA stenosis.

Genetic analysis of *RNF213* revealed wild-type p.R4810K, and no other rare variants were identified.

All genetic analyses were conducted with the approval of the institutional ethics committee (G2016-009) with written informed consent of patients and their families.

## Discussion

From our two cases, we have learned several lessons. (1) In patients with moyamoya disease, VA/BA stenosis accompanying shrinkage of outer diameter (i.e., negative remodeling) can develop or progress during follow-up after the initial diagnosis and may require revascularization surgery. (2) Even with serial follow-up using MRA, VA/BA stenosis can be missed if we do not consider it and may fall outside the image acquisition range. (3) The *RNF213* p.R4810K variant or other rare variants may not adequately explain the occurrence of VA/BA stenosis in this disease.

While several studies have reported VA/BA stenosis at initial diagnosis in patients with moyamoya disease, de novo development or progression of VA/BA stenosis has not been well established in the literature. An earlier report from Japan in 1984 indicated an incidence of 3% [9], while a report from China in 2016 reported an incidence of 1.5% [10]. Among patients with pediatric-onset moyamoya disease treated at our institution, VA/BA stenoses were present at initial diagnosis in 1.6% (2/129 cases) [13]. One recent study has shown that patients with moyamoya disease induced by rare variants in the ANO1 gene frequently developed stenosis in the BA and P1 portions of the PCA [15]. However, these studies only reported cases with VA/BA stenosis at initial diagnosis and did not include cases with de novo VA/BA stenosis during follow-up. Previous reports of de novo development of VA/BA lesions during follow-up were limited to the case reports of VA dissection [16], thrombotic occlusion of BA [17], and unilateral vertebral artery stenosis induced by rotational VA syndrome [18].

Our case 1 patient showed sudden occlusion of the stenosed portion of the bilateral VAs and cerebellar ischemic stroke following the revascularization surgery. We speculate this phenomenon was likely induced by hemodynamic changes associated with general anesthesia and the postoperative period, as some studies reported sudden occlusion of ICAs after the surgery [19].

Although they had VA/BA stenosis, both patients lacked ischemic stroke in the cerebellum or brainstem. Case 1 did not have any symptoms suggesting VA stenosis before developing postoperative stroke, and case 2 only developed mild symptoms that could be explained as vertebrobasilar insufficiency, and both patients lacked hemodynamic insufficiency in preoperative perfusion studies. In case 1, mild stenosis might not be severe enough to cause hemodynamic insufficiency. In case 2, slow progression of stenosis might allow the development of leptomeningeal collaterals from posterior inferior cerebellar arteries to anterior inferior cerebellar arteries that compensate for the decrease of antegrade flow. Diagnosing VA/BA stenosis might be difficult without a strong suspicion if it does not cause any symptoms or only symptoms of vertebrobasilar insufficiency. Therefore, routine follow-up imaging studies seem important to avoid missing this rare phenomenon.

Delayed recognition of progressive VA/BA stenosis in our patients was likely induced by the lack of MRA acquisition range to cover the VA/BA portions. In our institution, we evaluated six vessels, including bilateral VAs and BA at the initial catheter angiography, and evaluated extracranial VAs, ICAs, external carotid arteries (ECAs), and common carotid arteries (CCAs), as well as renal arteries. Nevertheless, after the initial diagnosis and during the follow-up, the MRA protocol for patients with moyamoya disease prioritized the vertex area to cover postoperative collaterals frequently developed in the vertex area and to visualize periventricular anastomosis, a well-known risk factor of subsequent hemorrhage in moyamoya disease [20]. It was difficult to cover both the lower portion and the vertex because the scanning time for MRA was limited due to the routine acquisition of cerebral blood flow maps using the arterial spin labeling method [11]. After experiencing these patients, we have started to check the lower portions of VA/BA regularly (roughly once in five years) for stable patients receiving annual MRI scans. Currently, after upgrading our MRI scanners, our MRA protocols cover a wider range from the distal VAs to the vertex, ensuring that BA stenosis will not be missed. Nevertheless, the proximal portions of the VAs may still be overlooked in routine protocols, depending on the cranial size of the patients. Perhaps, even if not every time, we should consider adding neck MRA to assess the proximal portions of the VAs, to check for possible de novo VA stenosis. Careful assessment of the VA/BA, as well as the arteries of the circle of Willis, is recommended during follow-up imaging so as not to miss stenosis in arteries outside the circle of Willis.

We had speculated that *RNF213* p.R4810K variant or other rare variants of this gene were related to VA/BA stenosis, but our cases suggested that RNF213 is not sufficient to explain this rare phenomenon. A previous study evaluated patients with intracranial atherosclerotic stroke and found that the outer diameter of non-stenosed BA was smaller in patients with* RNF213* variant compared to patients without it [7], so we had thought *RNF213* variants may relate to VA/BA stenosis with shrinkage of outer diameter. However, case 2 showed BA stenosis with shrinkage of outer diameter, or negative remodeling, which strongly indicated moyamoya disease [8], but did not carry any variant in *RNF213*. Additionally, among two patients in our institute with VA/BA stenoses at initial diagnosis, one carried a heterozygous *RNF213* p.R4810K variant, while the other had no variant in *RNF213*. Although a rare variant has been found to aggravate the clinical course of patients with moyamoya disease [13], neither patient had a rare variant of *RNF213*.

A recent study suggested that embryological differences may explain the lower incidence of VA/BA involvement in moyamoya disease. While anterior circulation arteries (ACA, MCA, and P2-4 of PCA) are derived from the primitive ICA, the posterior circulation arteries (VA/BA and P1 of PCA) originate from the longitudinal neural dorsal plexus and intersegmental arteries [10]. Moreover, vascular smooth muscle cells in the anterior circulation arteries are derived from the neural crest, whereas those in the posterior circulation arteries originate from mesodermal cells [15]. VA/BA stenosis in our patients might be induced by some abnormalities in mesodermal cells. However, direct evidence supporting embryological differences as a major factor remains lacking. Embryologic origin theory may not explain the more frequent involvement of renal, coronary, and pulmonary arteries in patients with moyamoya disease [21]. Perhaps other genetic interactions or environmental factors triggering inflammation or infection may contribute to this rare phenomenon. Longitudinal imaging follow-up together with 3D high-resolution vessel wall imaging [7] as well as genetic and epigenetic evaluation may clarify the mechanisms underlying VA/BA stenosis in moyamoya disease.

## Conclusions

VA/BA stenosis can develop or progress during follow-up in patients with moyamoya disease. Therefore, careful assessment of the VA/BA, as well as the arteries of the circle of Willis, is recommended during follow-up of these patients. *RNF213* gene variant appears insufficient to explain this rare phenomenon, but the small number of cases limits the plausibility of this hypothesis. Further accumulation of cases with longitudinal imaging follow-up up together with 3D high-resolution vessel wall imaging and genetic evaluation, ideally by a multicenter registry, is required to unravel the pathophysiology of this condition.
